# Surgically Enucleated Gastrointestinal Tumor of the Rectovaginal Septum

**DOI:** 10.7759/cureus.5019

**Published:** 2019-06-27

**Authors:** Brian H Le, Jasmine Nguyen, Anna Bossert, Tonie Crandall, Bernice Robinson-Bennett

**Affiliations:** 1 Pathology, Reading Hospital-Tower Health, West Reading, USA; 2 Medicine, Philadelphia College of Osteopathic Medicine, Philadelphia, USA; 3 Obstetrics and Gynecology, Reading Hospital, West Reading, USA; 4 Gynecologic Oncology, Reading Hospital, West Reading, USA

**Keywords:** gastrointestinal stromal tumor, pathology, rectovaginal septum

## Abstract

The rectovaginal septum is a rare location for gastrointestinal stromal tumors (GIST) to occur. When such is the case, the question arises as to whether the lesion, which is morphologically and immunophenotypically identical to its gastrointestinal counterpart, should be referred to as an extragastrointestinal stromal tumor (EGIST). A 77-year-old, gravida 4, para 4004 post-menopausal female with an unremarkable gynecologic history presented with brown vaginal discharge. On examination, a 4 to 5-cm nodule was palpated along the rectovaginal septum. Ultrasound revealed a 4.8-cm circumscribed, solid mass with internal blood flow located posterior and inferior to the cervix. At laparoscopy, the uterus and adnexae were deemed to be normal for age, without gross pathologic abnormalities. The nodule was resected in an enucleation procedure; subsequent histopathologic examination revealed a low-grade, spindled cell neoplasm with diffuse immunoreactivity for CD117 (cKit) and DOG1, diagnostic of GIST. Further molecular testing elucidated a mutation in exon 9 of the Kit gene. A decision was made by the patient for close observation; there is no clinical or radiographic evidence of recurrence one year after initial diagnosis.

## Introduction

Gastrointestinal stromal tumors (GIST), as the most common mesenchymal tumor of the gastrointestinal (GI) tract, typically originate from the tubal wall and extend intraluminally towards the mucosa, outwardly towards the serosal surface, or even in both directions [1-3]. They may also arise from extravisceral locations such as the omentum, mesentery, and retroperitoneum [4]. Those originating from the rectovaginal septum are especially rare and in such scenarios, raise a question regarding terminology, specifically as to whether the designation of extragastrointestinal stromal tumor (EGIST) is more appropriate [5-6]. Assessment for risk of the progressive disease relies on parameters that include tumor location, size, and mitotic rate, while molecular analysis facilitates prediction of response to targeted therapy [7-13]. This case illustrates nuances in the pathologic diagnosis and clinical management of a GIST (EGIST) arising from the rectovaginal septum.

## Case presentation

**Figure 1:**
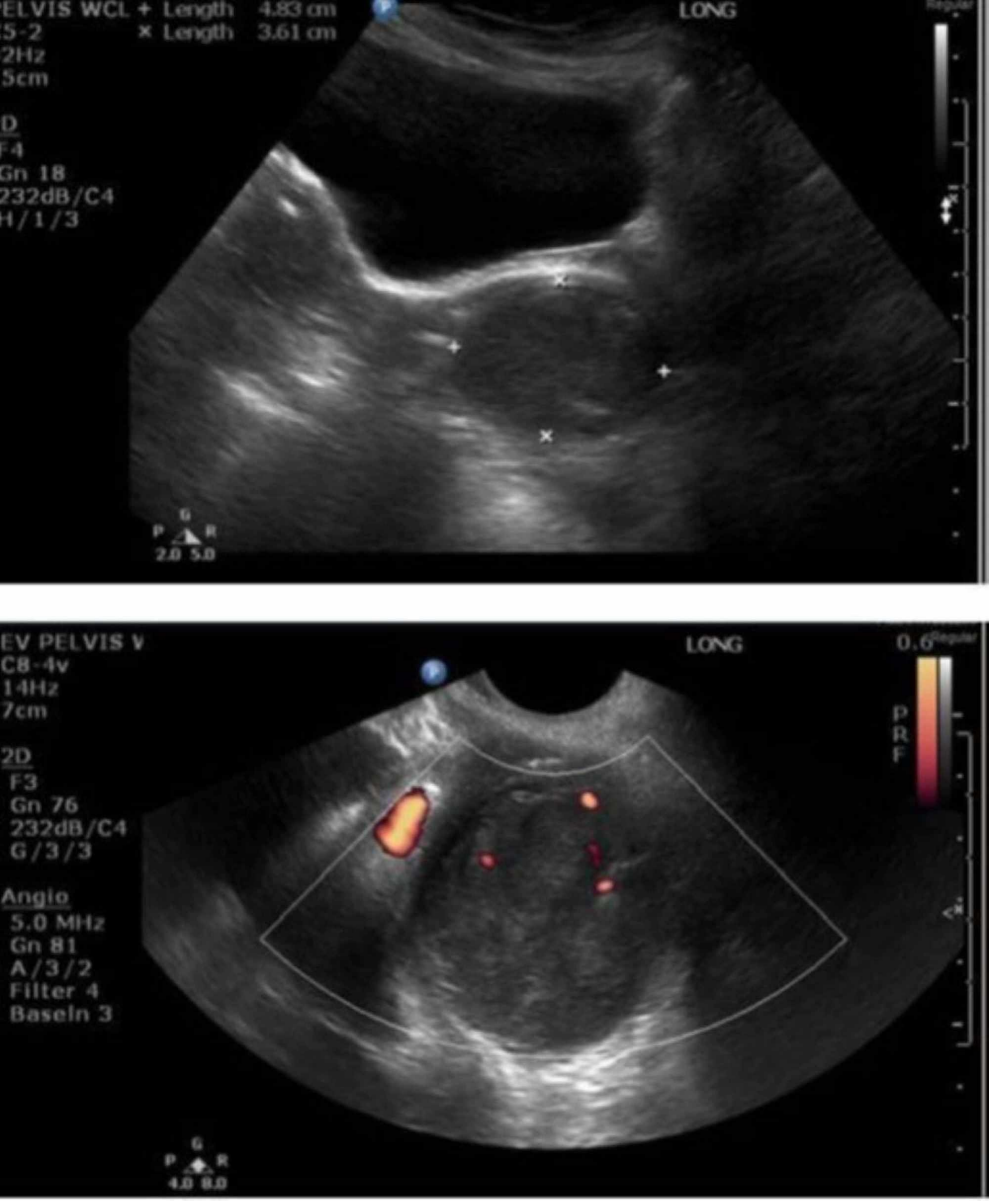
Transvaginal ultrasound with Doppler, showing a 4.8 x 4.4 x 3.6-cm solid mass lesion with internal blood flow, located posterior and inferior to the cervix

A 77-year-old, gravida 4, para 4004, post-menopausal female presented with brown vaginal discharge. Her gynecologic history had been essentially unremarkable, with negative Pap Tests, and negative testing for high-risk human Papillomavirus. Physical examination revealed atrophic external genitalia. A mobile, 4 to 5-cm nodule was palpated along the rectovaginal septum, with a muscle-like consistency and texture. Pelvic ultrasound showed a uterus and ovaries with measurements as expected for a post-menopausal state. In addition to a 2.5-cm calcified uterine fibroid, a 4.8-cm solid mass with internal blood flow was identified posterior and inferior to the cervix (Figure 1). 

**Figure 2:**
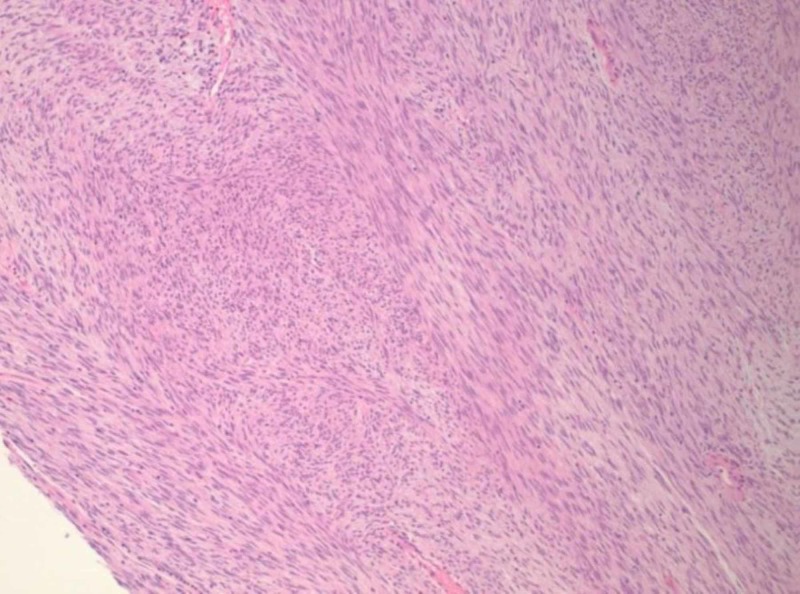
H&E stained section of the tumor showing a cellular proliferation with a fascicular architectural configuration (100x original magnification)

**Figure 3:**
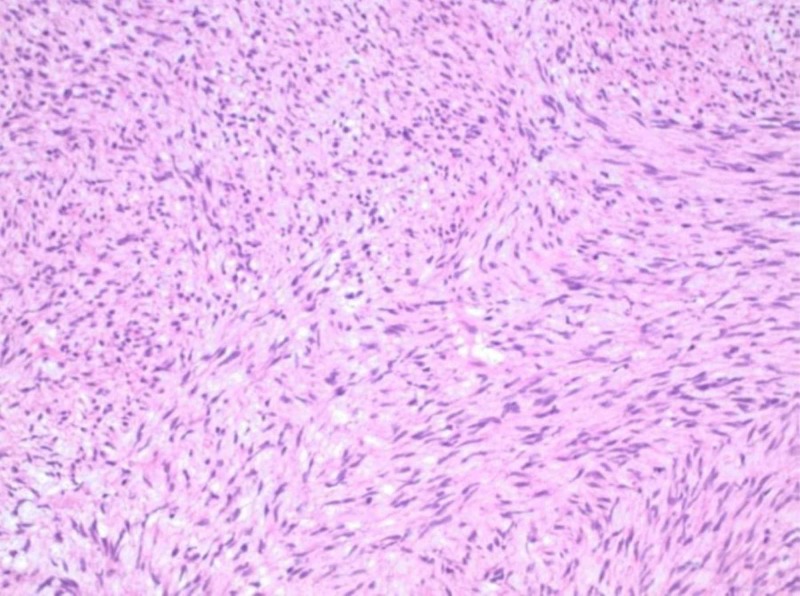
A proliferation of monotonous, bland-appearing spindled cells (200x original magnification)

**Figure 4:**
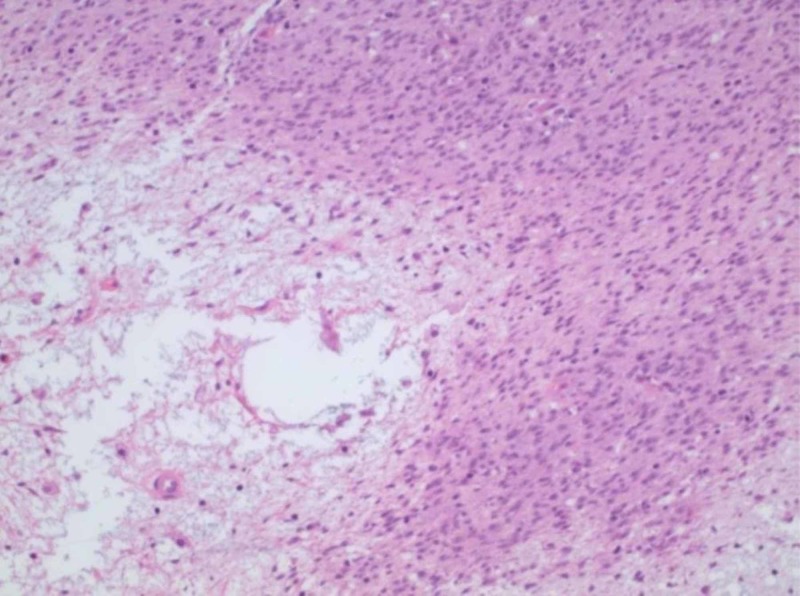
A rare focus showing microscopic necrosis (200x original magnification)

At subsequent laparoscopy, no intrapelvic abnormalities were seen. The previously identified mass occupying the rectovaginal space remained palpable; an enucleation of this mass was performed, yielding a 4.2 x 4.0 x 3.0-cm red-tan, circumscribed nodule with tan, whorled cut surfaces. Histologic examination demonstrated the proliferation of bland-appearing, spindled cells with a fascicular architectural configuration (Figures 2, 3). Few mitotic figures were identified, estimated at 3-4 per 5 mm^2^; a rare focus of necrosis was observed (Figure 4).

**Figure 5:**
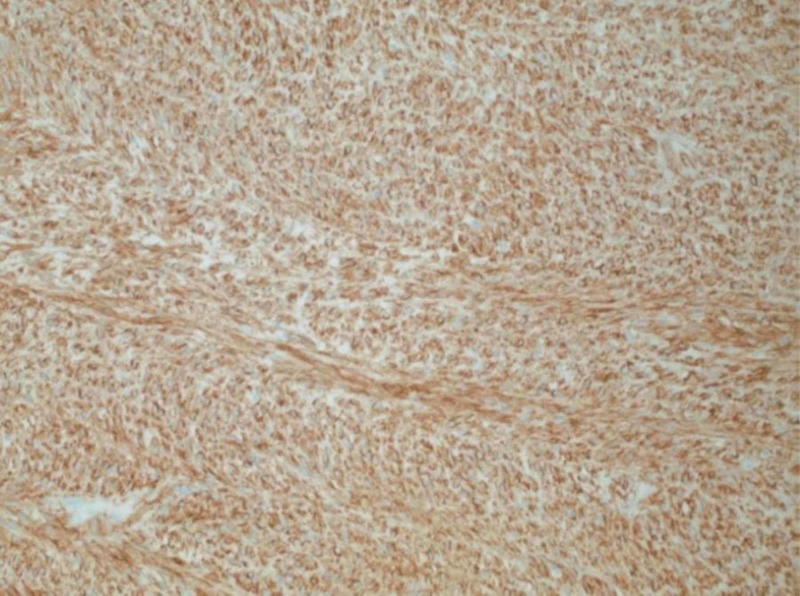
Diffuse immunoreactivity for cKit (CD117) in neoplastic cells (200x original magnification)

**Figure 6:**
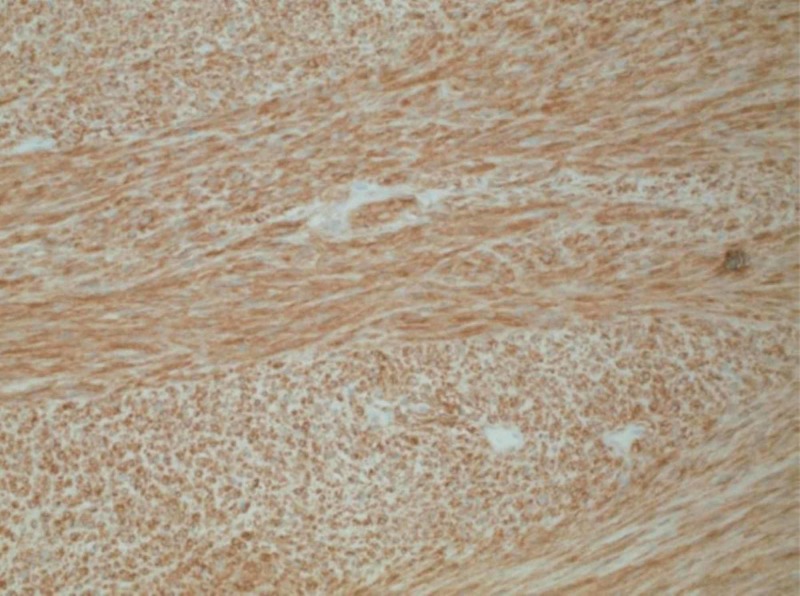
Diffuse immunoreactivity for DOG1 in neoplastic cells (200x original magnification)

In an attempt to further characterize the lesion, a panel of immunohistochemical preparations was performed. Neoplastic cells showed diffuse reactivity for vimentin, c-Kit (Figure 5), and DOG1 (Figure 6). Immunohistochemistry for pan-cytokeratin, desmin, S-100 protein, and SOX10 was interpreted as negative; smooth muscle actin accentuated background vascular endothelium. The global morphologic features, in correlation with immunophenotype, were diagnostic of GISTs. Further molecular testing elucidated a mutation in exon 9 of the Kit gene.

The patient presented with various management options, including imatinib versus sunitinib therapy, rectal resection, and active surveillance. She chose to pursue close active surveillance, with pelvic MRI every three months over an interval of two years. At approximately one year following surgical enucleation and close surveillance, there is no clinical or radiographic evidence of recurrence.

## Discussion

GISTs are the most common mesenchymal tumors of the gastrointestinal tract, typically arising in the wall of the stomach or small intestine [1-3]. Rare cases are identified outside of the intestinal tract; such cases may be collectively referred to as EGISTs, demonstrating similar morphologic features and immunophenotypes to GISTs [4]. Occurrence in the rectovaginal septum is rare, with only a few reported cases in the literature [5-6,14-17]. This scenario illustrates an example of a GIST arising within the vicinity of the rectum, but without a definitive apparent connection to, or an origin from the rectum.

The histologic differential diagnosis of GIST is broad and includes a sarcomatoid carcinoma, a peripheral nerve sheath tumor such as schwannoma, and leiomyoma versus leiomyosarcoma. Immunohistochemistry is especially useful in diagnostic distinction, as GISTs are generally expected to demonstrate reactivity for c-Kit (CD117) [18-19]. While 95% of GISTs express c-Kit (CD117), approximately 95% also express DOG1 [20]. DOG1 has been proven to be a sensitive and specific marker for the GISTs, including cases of extragastrointestinal and metastatic lesions [20]. In this particular case, the absence of reactivity for markers associated with muscle differentiation, namely desmin and smooth muscle actin, argues against leiomyoma and leiomyosarcoma, while non-reactivity for pan-cytokeratin renders an epithelial tumor unlikely. Additionally, non-reactivity for S-100 protein argues against schwannoma. The differential diagnostic possibility of GIST is confirmed by diffuse reactivity for c-Kit and DOG1; this is further substantiated by detection of a mutation in exon 9 of the Kit gene.

As remote recurrences of GISTs may be encountered years after excision, most regard this tumor as having at least some potential for metastasis [1]. Guidelines for risk assessment rely on tumor location (gastric, duodenum, jejunum/ileum, and versus rectum), tumor size (<2 cm, 2-5 cm, 5-10 cm, >10 cm), and mitotic rate (five or fewer per 5 mm^2^ versus >5 per 5 mm^2^) [8]. In this particular case, a tumor size of 4.2 cm and a mitotic rate of less than five per 5 mm^2^ would estimate the risk of progressive disease of approximately 8.5%; if the lesion was believed to originate from the rectum, the closest gastrointestinal anatomical site [8]. However, without evidence of a direct connection to a luminal gastrointestinal structure, it is difficult to ascertain the true risk of recurrence and of progressive disease. This case is further nuanced by the fact that enucleation was believed to have completely contained the tumor, which showed no direct connection to a bowel wall at the time of surgery. As such, there was an absence of a non-neoplastic tissue interface to document negative margins. Consequently, there would not be a specific tissue target to further resect. As a result, additional surgery to the area would introduce additional unnecessary morbidity.

It is estimated that approximately 85% of GISTs demonstrate mutations in the KIT gene, while approximately 10% harbor PDGFRA gene mutations; the remaining may show mutations in both genes [10-11]. Those with mutations in exon 9 are expected to have better relapse-free survival and overall survival and are more likely to respond to second-line targeted therapy than those with other mutational profiles [12-13]. Molecular analysis of the current case demonstrated a mutation in exon 9 of the KIT gene, with no evidence of mutation in the AKT1, BRAF, CTNNB1, FGFR1, FGFR2, FGFR3, PDGFRA, or SRC genes. With this more favorable mutational profile, and, in consideration of the patient’s age, it was believed that should evidence of clinical or radiographic recurrence be detected during a course of active surveillance, therapy with imatinib could be considered and initiated without losing any window of opportunity for optimal survival. In consideration of the options presented, the patient chose a course of close observation with pelvic MRI every three months over two years. At approximately one year follow-up, there is no evidence of clinical or radiographic recurrence.

## Conclusions

Although GISTs rarely arise in the rectovaginal septum, it should be considered in the differential diagnosis of a spindled cell lesion when encountered in such location. The absence of a direct mural connection to the tubular intestinal tract raises the possibility that such lesion should be referred to as "extragastrointestinal stromal tumor", or EGIST.

## References

[1] Fletcher CD, Berman JJ, Corless C (2002). Diagnosis of gastrointestinal stromal tumors: a consensus approach. Hum Pathol.

[2] Miettinen M, Furlong M, Sarlomo-Rikala M, Burke A, Sobin LH, Lasota J (2001). Gastrointestinal stromal tumors, intramural leiomyomas, and leiomyosarcomas in the rectum and anus: a clinicopathologic, immunohistochemical, and molecular genetic study of 144 cases. Am J Surg Pathol.

[3] Miettinen M, Lasota J (2001). Gastrointestinal stromal tumors: definition, clinical, histological, immunohistochemical, and molecular genetic features and differential diagnosis. Virchows Arch.

[4] Reith JD, Goldblum JR, Lyles RH, Weiss SW (2000). Extragastrointestinal (soft tissue) stromal tumors: an analysis of 48 cases with emphasis on histologic predictors of outcome. Mod Pathol.

[5] Lee YH, Chong GO, Hong DG (2015). Is gastrointestinal stromal tumor (GIST) originating from the rectovaginal septum GIST or extra-GIST (EGIST)? A case report with literature review. Eur J Gynaecol Oncol.

[6] Zhang W, Peng Z, Xu L (2009). Extragastrointestinal stromal tumor arising in the rectovaginal septum: report of an unusual case with literature review. Gynecol Oncol.

[7] Miettinen M, Makhlouf H, Sobin LH, Lasota J (2006). Gastrointestinal stromal tumors of the jejunum and ileum: a clinicopathologic, immunohistochemical, and molecular genetic study of 906 cases before imatinib with long-term follow-up. Am J Surg Pathol.

[8] Miettinen M, Lasota J (2006). Gastrointestinal stromal tumors: pathology and prognosis at different sites. Semin Diagn Pathol.

[9] Heinrich MC, Corless CL, Demetri GD (2003). Kinase mutations and imatinib response in patients with metastatic gastrointestinal stromal tumor. J Clin Oncol.

[10] Heinrich MC, Corless CL, Duensing A (Science). PDGFRA activating mutations in gastrointestinal stromal tumors.

[11] Heinrich MC, Corless CL, Blanke CD (2006). Molecular correlates of imatinib resistance in gastrointestinal stromal tumors. J Clin Oncol.

[12] Demetri GD (2002). Targeting the molecular pathophysiology of gastrointestinal stromal tumors with imatinib: mechanisms, successes, and challenges to rational drug development. Hematol Oncol Clin North Am.

[13] Demetri GD, van Oosterom AT, Garrett CR (2006). Efficacy and safety of sunitinib in patients with advanced gastrointestinal stromal tumour after failure of imatinib: a randomised controlled trial. Lancet.

[14] Nasu K, Ueda T, Kai S, Anai H, Kimura Y, Yokohama S, Miyakawa I (2004). Gastrointestinal stromal tumor arising in the rectovaginal septum. Int J Gynecol Cancer.

[15] Vazquez J, Perez-Pena M, Gonzalez B, Sanchez A (2012). Gastrointestinal stromal tumor arising in the rectovaginal septum. J Low Genit Tract Dis.

[16] Hara M, Takayam S, Arakawa A, Sato M, Nagasaki T, Takeyama H (2012). Transvaginal resection of a rectal gastrointestinal stromal tumor. Surg Today.

[17] Lam MM, Corless CL, Goldblum JR, Heinrich MC, Downs-Kelly E, Rubin BP (2006). Extragastrointestinal stromal tumors presenting as vulvovaginal/rectovaginal septal masses: a diagnostic pitfall. Int J Gynecol Pathol.

[18] Hornick JL, Fletcher CD (2002). Immunohistochemical staining for KIT (CD117) in soft tissue sarcomas is very limited in distribution. Am J Clin Pathol.

[19] Miettinen M, Sobin LH, Sarlomo-Rikala M (2000). Immunohistochemical spectrum of GISTs at different sites and their differential diagnosis with a reference to CD117 (KIT). Mod Pathol.

[20] Fatima N, Cohen C, Siddiqui MT (2011). DOG1 utility in diagnosing gastrointestinal stromal tumors on fine-needle aspiration. Cancer Cytopathol.

